# Cloning of the Heat Shock Protein 60 Gene from the Stem Borer, *Chilo suppressalis*, and Analysis of Expression Characteristics Under Heat Stress

**DOI:** 10.1673/031.010.10001

**Published:** 2010-07-09

**Authors:** Ya-Dong Cui, Yu-Zhou Du, Ming-Xing Lu, Cheng-Kui Qiang

**Affiliations:** ^1^ Institute of Applied Entomology, Yangzhou University, Yangzhou, 225009, China; ^2^ Department of Life Science, Fuyang Teachers College, Fuyang, 236032, China; ^3^ Department of Agriculture and Landscape Engineering, Xuzhou Higher Vocational School of Bioengineering, Xuzhou, 221006, China

**Keywords:** Lepidoptera, Hsp60, RACE, haemocytes, temperature

## Abstract

Heat shock protein 60 is an important chaperonin. In this paper, *hsp60* of the stem borer, *Chilo suppressalis* (Walker) (Lepidoptera: Pyralidae), was cloned by RT-PCR and rapid amplification of cDNA end (RACE) reactions. The full length cDNA of *hsp6°C*onsisted of 2142 bp, with an ORF of 1719 bp, encoding 572 amino acid residues, with a 5'UTR of 158 bp and a 3'UTR of 265 bp. Cluster analysis confirmed that the deduced amino acid sequence shared high identity with the reported sequences from other insects (77%–86%). To investigate whether *hsp60* in *C. suppressalis* responds to thermal stress, the expression levels of *hsp60* mRNA in larval haemocytes across temperature gradients from 31 to 39°C were analysed by real-time quantitative PCR. There was no significant difference for *hsp60* expression from 28 to 31°C. he temperatures for maximal induction of *hsp60* expression in haemocytes was close to 36°C. Hsp60 expression was observed by using flow cytometry. These results revealed that thermal stress significantly induced *hsp60* expression and Hsp60 synthesis in larval haemocytes, and the expression profiles of Hsp60 at the mRNA and protein levels were in high agreement with each other from 33 to 39°C.

## Introduction

Heat shock proteins (Hsp) are a family of proteins that help organisms to modulate stress response and protect organisms from environmentally induced cellular damage. They usually act as molecular chaperones, promoting correct refolding and preventing aggregation of denatured proteins (Johnston et al. 1998; Feder and Hofmann 1999). On the basis of molecular weight and homology of amino acid sequences, Hsp can be divided into several families including Hsp90, 70, 60, 40 and small Hsp (Feder and Hofmann 1999; Sørensen et al. 2003).

Hsp60 is mostly located in mitochondria of eukaryotic cells (Gatenby et al. 1991). Under normal conditions, Hsp60 operates the bending and assembling of enzymes and other protein complexes related to energy metabolism; under adverse environmental conditions the synthesis of Hsp60 increases and the protein then renatures damaged proteins to restore their biological activity (Buchner et al. 1991; Cheng et al. 1989; Ostermann et al. 1989; Martin et al. 1991). As a molecular chaperone, Hsp60 helps protect against protein aggregation (Sanders et al. 1992), and in the transport of proteins from cytoplasm to organelles (Fink 1999).

Molecular analysis of thermal stress has been extensively studied in *Drosophila melanogaster* and shows various responses of each Hsp at the transcriptional and translational level (Hoffman et al. 2003; Sørensen et al. 2007). Recently, research has been extended to several other insect species that are important in agricultural, medical and industrial fields (Yocum 2001; Chen et al. 2005, 2006; Sonoda et al. 2006; Huang and Kang 2007; Wang et al. 2007; Kim et al. 2008). The stem borer, *Chilo suppressalis* (Walker) (Lepidoptera: Pyralidae) is one of the most serious pests of rice. This pest has been widely distributed in all rice fields of China, and is constantly adapting to its environment. In the present study, the full-length cDNA of *C. suppressalis hsp60* was cloned. In addition, the expression profiles of Hsp60 in larvae haemocytes from *C. suppressalis* at mRNA and protein levels were analysed by using real-time quantitative PCR and flow cytometry across temperature gradients from 31 to 39°C.

## Materials and Methods

### Insect rearing and Isolation of haemocytes

The larvae of *C. suppressalis* were initially collected from the paddy fields in suburbs of Yangzhou City, China. The rice stem borers were reared using a method described by Shang et al. (1979) at 28 ± 1°C, 16:8 L:D and RH >80%. Hatched larvae were fed on rice seedlings until larvae reached the 5th stadium.

Larvae from each experimental group were washed two times with distilled water and were anaesthetised by chilling on ice. The proleg was then cut off and haemolymph was collected directly into 1ml Eppendorf tubes containing 200 µl cold PBS. The haemocytes were separated from the haemolymph by centrifugation for 5 min at 800 g at 4°C. Sedimented haemocytes were washed twice with PBS and used for RNA extraction.

### RNA extraction and cDNA synthesis

Total RNA from the haemocytes was isolated using TRIzol reagent (Sangon, www.sangon.com). Single-stranded cDNA was synthesized from 1 µg of RNA with the MBI RevertAid First Strand cDNA Synthesis Kit (MBI Fermentas, www.fermentas.com) according to the manufacturer's instructions. Single-stranded cDNA for 3′-Rapid amplification of cDNA ends (3′-RACE) and 5′-RACE experiments was synthesized from 1 µg of RNA using the TaKaRa RACE cDNA Amplification Kit (TaKaRa,_www.takarabio.co.jp) according to the manufacturer's instructions.

### Degenerate PCR for isolation of *hsp60* fragments

The partial clones of *hsp60* from *C. suppressalis* were amplified by PCR using primer sets: P1, P2 (Table 1). Pairs of primers were designed using consensus amino acid of insect Hsp60. PCR was carried out using 2 µl cDNA, 15 pmol of each primer, 10 nmol of each deoxynucleoside triphosphate (dNTP), and 1 unit of *Taq* DNA polymerase (TaKaRa) in the supplied buffer giving a final concentration of 2.0 mM MgCl_2_ in 25 µl. Cycle conditions were as follows: initial denaturation at 94°C for 4 min; 3°Cycles of 94°C for 40 s, 55°C for 40 s, and 72°C for 1 min; final extension at 72°C for 10 min. Amplification products were purified from 1% agarose gels using a gel extraction kit (BioTeke, www.biotek.com). The purified fragment was cloned into the pMD-18T vector (TaKaRa) and sequenced.

**Table 1.**
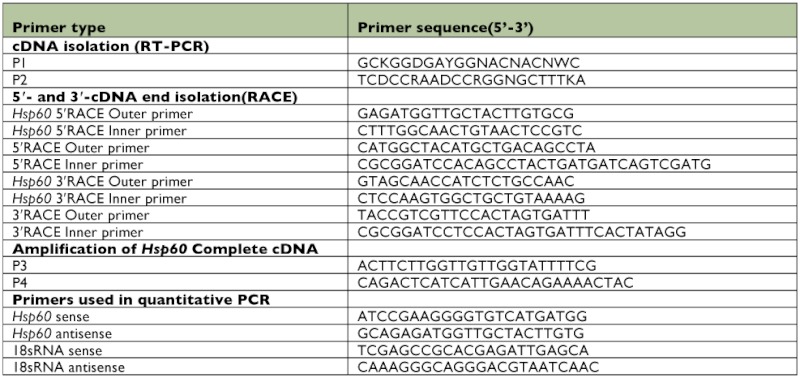
Primer sequences used in the cDNA cloning and real-time PCR

### 3′-RACE PC

Two semi-nested 3′-RACE reactions were conducted on 2 µl of the *C. suppressalis* 3′RACE-ready cDNA. In the first reaction, a sense gene-specific primer designed from P1 and P2 PCR product sequence (*hsp60* 3′RACE outer primer; Table 1) and an antisense 3′-RACE outer primer (Table 1; TaKaRa) were used. The 3′-RACE inner PCR was performed using the *hsp60* 3′-RACE inner primer (Table 1) and the antisense 3′-RACE inner primer (Table 1; TaKaRa). The 50-µl amplification mix was prepared according to the TaKaRa cDNA protocol using the *La Taq* polymerase mix (TaKaRa). The outer and inner PCR were performed using the following conditions: 3 min at 94°C, followed by 25 cycles of 40 s at 94°C, 40 s at 55°C, and 2 min at 72°C and finishing with chain extension at 72°C for 10 min.

### 5′-RACE PCR

Two semi-nested 5′-RACE reactions were conducted on 2 µl of *C. suppressalis* 5′RACE-ready cDNA. In the first reaction, a sense gene-specific primer designed from the sequences obtained following 3′-RACE outer and inner PCR amplifications of *hsp60* (*hsp60* 5′-RACE outer primer; Table 1) and an antisense 5′-RACE outer primer (Table 1; TaKaRa) were used. The 5′-RACE inner PCR was performed using the *hsp60* 5′-RACE inner primer (Table 1) and the antisense 5′RACE inner primer (Table 1; TaKaRa). PCRs were performed as described for the 3′-RACE PCR.

### Cloning and sequencing

After gel extraction, the 3′- and 5′-RACE fragments were cloned into a pMD-18T vector (TaKaRa). Recombinant plasmids were isolated using the Plasmid Mini kit (BioTeke), and sequenced. The full-length of the sequence assembled by RACE was verified by sequencing the fragment amplified from the primers P3, P4 (located at 5′ UTR and 3′UTR) (Table 1) and subjected to homology analysis.

### Amino acid sequence comparisons and phylogenetic analysis

Database searches were performed with the BLAST program (NCBI-BLAST network server). The open reading frame was identified using ORF Finder (http://www.ncbi.nlm.nih.gov/gorf/gorf.html) and the amino acid molecular weight was calculated by the SWISS-PROT (ExPASy server) program ‘Compute pI/Mw’ (http://au.expasy.org/). Sequence alignment and homology analysis was performed using Clustal X. A phylogenetic tree (neighborjoining method) was then constructed with 1000 bootstrap replicates using MEGA version 3.1 based on the deduced amino acid sequence of *C. suppressalis* Hsp60 as well as the known sequences of fourteen other insect species.

### Real-time quantitative PCR

Fifteen fifth-instar larvae in three replicates were randomly selected from each experimental group and exposed to 31, 33, 36 or 39°C for 2 h with 28°C as control. Each treatment was repeated three times.

The haemocytes from control and treated groups were collected as above. The collected haemocytes were immediately used for RNA extraction, and cDNA was synthesized according to the methods described above. 18S rRNA gene in *C. suppressalis* was cloned (GQ265912) and used as the housekeeping gene. Based on the cDNA sequences of *C. suppressalis hsp60* and the 18S rRNA gene sequences, primer pairs (*hsp60* sense / *hsp60* antisense and 18S sense / 18S antisense) for real-time PCR were designed (Table 1). The PCR reactions were performed in a 20 µl total reaction volume including 10 µl of 2 × SYBR® Premix EX Taq™ master mix (TaKaRa), 5 µM each of primers (Table 1) and 1 µl cDNA templates. They were carried out on the ABI Prism 7000 Sequence Detection System (Applied Biosystems, www.appliedbiosystems.com). The thermal cycler parameters were as follows: 10 s at 95°C, then 4°Cycles of 5 s at 95°C, 20 s at 55°C and 20 s at 72°C.

For each amplification, the reaction was carried out in 3 replicates, from which mean threshold cycle (CT) values plus standard deviations were calculated. Relative transcripts of *C. suppressalis hsp6°C*DNA amounts were calculated applying the 2^-ΔΔ^C^T^ method (Livak and Schmittgen 2001).

### Flow cytometric determination of Hsp60 levels

The larval treatment was carried out as described above. Each temperature treatment was repeated three times. The haemocytes were harvested as described above. The extent of Hsp60 levels in larvae haemocytes was investigated following the method of Shen and Zhou (2002).

**Figure 1.**
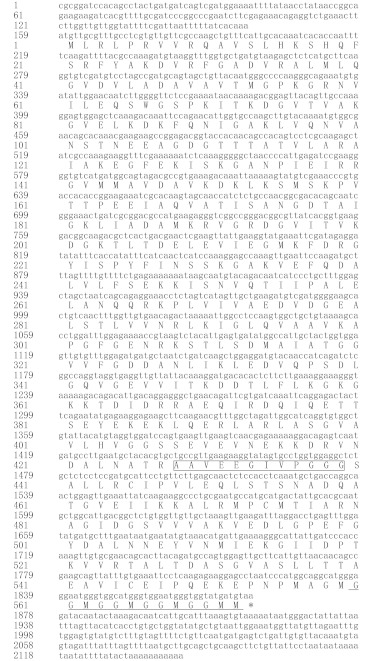
The complete cDNA sequence and predicted amino acid sequence of *Hsp60* of *Chilo suppressalis*. A classical mitochondrial Hsp60 signature is shown in box, and a typical GGM repeat motif at the C terminus is underlined. The stop codon is marked with an asterisk. High quality figures are available online.

The collected haemocytes were fixed in 4% paraformaldehyde used for analysis of Hsp60 levels. Each sample was divided into two groups, one group for positive treatment, another as a negative baseline control. Positive treatment was performed as follows: the fixed haemocytes were centrifuged for 5 min at 800 g and washed twice with PBS. Subsequently, the haemocytes were permeabilized in PBS with Triton X-100 and incubated with the rabbit anti-Hsp60 polyclonal antibody (Boster, www.immunoleader.com/) (1:200) for 30 min at 18°C, and then centrifuged for 5 min at 800g and washed twice with PBS. Following treatment with the primary antibody, the haemocytes were incubated for 30 min at 18°C with FITC conjugated goat anti-rabbit secondary antibody (Boster) (1:300) and then centrifuged for 5 min at 800g and washed twice with PBS. Finally the haemocytes were resuspended in PBS for analysis. Negative baseline control procedure was similar to that of positive treatment except that the primary antibody incubation was eliminated.

Cellular fluorescence, reflecting cellular Hsp60 levels, was determined at 525 nm using flow cytometry (Becton Dickinson, www.bd.com). For each group, approximately 1000°Cells were analysed. Subsequently, the average fluorescence values of the positive treatment were divided by those of the negative baseline control, and the folds were used as the relative levels of Hsp60 from each sample.

### Statistical analysis

Data were expressed as mean values ± S.D. based on three separate experiments. Statistical analysis was carried out by Student's t-test.The asterisks denote statistical significance when compared with control: *p < 0.05; **p < 0.01.

## Results

### Sequence analysis

Degenerative primers based on a conserved region of *hsp60* were used to amplify the cDNAs derived from the haemocytes of *C. suppressalis*. A PCR product of 596 bp was obtained. The 596 bp amplified fragment was highly homologous to *hsp60*, and used to obtain 5′- and 3′ flanking sequences using RACE 5′- and 3′ RACE of *hsp60* produced fragments of 456 and 1142 bp. After assembly of the 2 sequences, a 2142 bp full-length cDNA sequence was obtained (Figure 1). This sequence contained a 158 bp 5′UTR, a 265 bp 3′UTR with a canonical polyadenylation signal sequence (AATAAA), and a poly (A) tail, as well as a single 1,719 bp open reading frame (ORF) encoding a polypeptide comprised of 572 amino acids with a molecular mass of 61014 Da and a pI of 5.69 (GenBank accession number: GQ265913).

**Figure 2.**
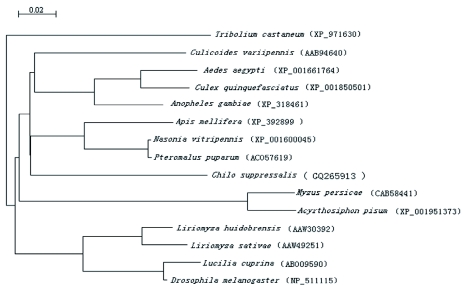
Phylogenetic tree based on amino acid sequences of mitochondrial *Hsp60* (constructed using the Clustal W programme). The GenBank accession numbers for amino acid sequence data were shown in the brackets. High quality figures are available online.

The deduced amino acid sequence of Hsp60 of *C. suppressalis* was highly similar to that of other insects including: *Culicoides variipennis* (86%), *Pteromalus puparum* and *Nasonia vitripennis* (85%), *Anopheles gambiae, Apis mellifera* and *Culex quinquefasciatus* (83%), *Drosophila melanogaster , Liriomyza sativae , Lucilia cuprina* and *Aedes aegypti* (82 %), *Liriomyza huidobrensis* (81%), *Tribolium castaneum* (79%), *Myzus persicae* (78%), *Acyrthosiphon pisum* (77%). Based on the amino acid sequences of Hsp60, a phylogenetic tree was constructed using the programs of CLUSTALX and MEGA2.1 (Figure 2).

### Real-time quantitative analysis of *hsp60* expression

**Figure 3.**
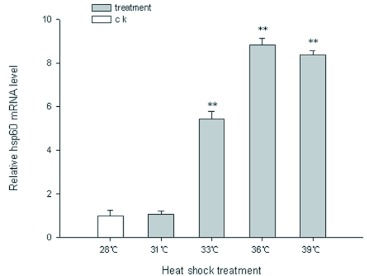
Expression analysis of *hsp60* mRNA of haemocytes from *Chilo suppressalis* under different heat-shock temperature by Real-time PCR. Values are means ± S.D. of three independent experiments in triplicate. **p < 0.05 vs. control, **p < 0.01 vs. control. High quality figures are available online.

To determine whether *hsp60* responds to heat treatments, fifth-instar larvae of *C. suppressalis* were kept at a target temperature (31, 33, 36 or 39°C) for 2h and expression levels were compared with levels observed in non heat-treated individuals at 28°C. As shown in Figure 3 *hsp60* mRNA was expressed at extremely low levels in the untreated groups (28°C). Under thermal stress, the baseline levels of *hsp60* mRNA were found to vary. A significant induction of *hsp60* was shown at 33, 36 and 39°C, reaching 5.61, 9.13 and 8.67 fold versus the control, respectively, and there was no significant difference for *hsp60* expression from 28 to 31°C. Interestingly, the degree of increase in the level of hsp60 reached a maximum at 36°C and then dropped at 39°C with increasing temperatures.

#### Hsp60 verification at protein levels

**Figure 4:**
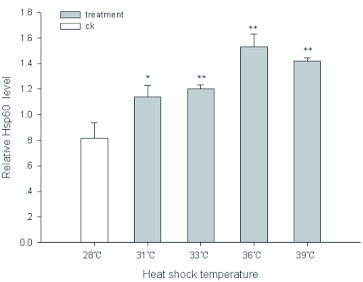
Expression levels of Hsp60 of haemocytes from *Chilo suppressalis* under different heat-shock temperature by using Flow cytometry. Values are means ± S.D. of three independent experiments in triplicate. *p < 0.05 vs. control. **p < 0.01 vs. control. High quality figures are available online.

To study the heat induction of Hsp60 at the translational levels, the expression of Hsp60 was determined by using a flow cytometer. Figure 4 shows that thermal stress significantly elevated the level of Hsp60 synthesis in larvae haemocytes. Compared with the control (28°C), the relative levels of Hsp60 increased to 1.40, 1.47, 1.88 and 1.74 fold at 31, 33, 36, and 39°C, respectively. These results revealed that the expression profiles of Hsp60 at the mRNA and protein levels are in high agreement with each other from 33 to 39°C.

## Discussion

Using a combination of RT-PCR and RACE techniques, the full-length *hsp6°C*DNA was cloned from haemocyts of *C. suppressalis*. The C-terminal repeats (GGM)_n_, which are a characteritic of mitochondrial Hsp60 (Tsugekils et al. 1992), are present in *C. suppressalis* Hsp60, indicating that the isolated gene is a mitochondrial *hsp60*. In addition, the lengths of the cDNA and the ORF, as well as the predicted protein size are similar to those of other Hsp60s. An ATP binding motif, which is highly conserved (Wong et al. 2004), was found in the deduced Hsp60 amino acid sequence. The great similarity in this region may indicate that among Hsp60s, the mechanism of coupling ATP hydrolysis to the substrate-refolding process is similar. Using the BLAST X programme of the NCBI website, the deduced amino acid sequence of Hsp60 of *C. suppressalis* showed high identity and similarity with known Hsp60s of other insect species (more than 77% similarity in all the matches). A phylogentic tree was constructed based on the full amino acid sequences of Hsp60 of the fifteen insect species in this study.

To find the temperatures for maximal induction of *hsp60* expression, the relative mRNA levels of *hsp60* in larvae haemocytes of *C. suppressalis* were quantified by real-time quantitative PCR at temperatures from 31 to 39°C. It was found that *hsp60* mRNA in larval haemocytes was expressed at extremely low levels in the control groups (under normal conditions). The baseline levels of *hsp60* mRNA were found to vary under heat stress. The results revealed that *hsp60* gene in larvae haemocytes was significantly upregulated with increasing temperatures, reached a maximum at 2h of exposure to a 36°C heat shock and then dropped at 39°C. However, after exposure at 39°C for 2h, *hsp60* mRNA levels exhibited a reduction in expression, indicating that transcription had decreased. Huang and Kang (2007) observed a similar response in the induction of heat shock in *L. sativae hsp60* using real-time quantitative PCR methods, and the expression of *L. sativae hsp60* was inhibited when temperatures were higher than 42.5°C for 1 h, which exceeds the tolerance limit of *L. sativae*. However, the majority of previous studies of inducible *hsp60s* expression was virtually undetectable by stress factors. For instance, the expression levels of mitochondrial *hsp60* are not influenced by heat or cold in *Trichinella spiralis* (Wong et al. 2004). Furthermore, the expression of *hsp60* does not respond to various types of stresses, such as H2O2 (Martinez et al. 2002), acidic and oxidative stress (Wong et al. 2004). In those studies, mRNA expression was monitored using Northern Blotting or semi-quantitative RTPCR, which is less sensitive than real-time quantitative PCR.

Hsp60 is known to function as a molecular chaperone in many species and is absolutely essential for the proper functioning of cells under normal and stress conditions (Lindquist 1986; Hemmingsen et al. 1988; Goloubinoff et al. 1989; Hartl 1996). In this study, we detected up-regulation of Hsp60 in haemocytes of *C. suppressalis* in adaptation to thermal stress. Hsp60 in haemocytes was found to be increased at 31°C while *hsp60* was not increased. The results indicate that the thermal responses of Hsp60 in the haemocytes at the mRNA and protein levels are high in agreement with each other from 33 to 39°C. Our findings also agree with those of Wheelock et al. (1999) for *B. plicatilis* in which Hsp60 response increased up to 3–4 fold when heat exposure occurred. Rios-Arana et al. (2005) also reported that Hsp60 was induced 2–4 fold in *P. patulus* exposed to heat. Other arthropod studies have compared expression levels at gene and protein levels. For example, protein levels of Hsp70 followed thermotolerance and reached the highest levels 49 h after heat hardening in adult *Orchesella cincta*, and the expression of *hsp70* messenger RNA reached a peak within the first 2 h and then sharply decreased after 6 h(Bahrndorffetal. 2009).

Mitochondria are essential eukaryotic organelles that serve as a site for many vital metabolic pathways and supply the cell with oxidative energy. Hsp60 plays a central role in the folding of newly imported and stress-denatured proteins (Martin et al. 1992; Martinus et al. 1995). As so, it was demonstrated that yeast containing mutated mt-Hsp60 do not grow at elevated temperatures (Cheng et al. 1989; Dubaquie et al. 1998) and show irreversible aggregation of a large number of newly imported proteins (Dubaquie et al. 1998). The higher level of Hsp60 expression induced by heat stress strengthens the idea that this protein has a significant role in the adaptation of various environmental conditions.

Some studies indicated that the induction of Hsp60 expression is tissue-specific. Lakhotia and Singh (1996) reported Hsp60 heat-induced expression in *D. melanogaster* larval Malpighian tubules following heat shock. A tissue-specific variation in the heat-induced expression of Hsp60 was also reported in grasshopper (*Spathosternum prasiniferum*), cockroach (*Periplanata americana*) and gram pest (*Heliothis armigera*) (Singh and Lakhotia 2000). The level of Hsp60 in *L. cuprina* was significantly enhanced upon heat shock in some tissues (Sunita et al. 2006). Moreover, over-expression of Hsp may cause some negative effects on growth, development, survival and fecundity (Krebs and Feder 1997; Krebs and Feder 1998; Huang et al. 2007), suggesting that the expression of Hsp may relate to physiological processes (Huang et al. 2007). Interestingly, the expression levels of Hsp60 in the haemocytes of *C. suppressalis* reached a maximum at 36°C and then declined at 39°C with increasing temperatures in the present study. Such a drop of Hsp60 may be due to approach of a threshold limit in the cell. A similar scenario was observed in an earlier study in which Hsp60 synthesis in *L. huidobrensis* were found to increase to a maximum at 42.5°C and then dropped when heat stress was enhanced (Huang and Kang 2007). Kristensen et al. (2002) also reported that inbred larvae of *Drosophila buzzatii* expressed more Hsp70 at high temperatures except at very high temperatures close to the physiological limit. This further supports the fact that Hsp plays a major protective role against cellular damage with high temperature exposure and yet may not be able to protect the cells beyond the threshold limit.

## Abbreviatons

Hsp: heat shock protein
